# Personalized tumor combination therapy optimization using the single-cell transcriptome

**DOI:** 10.1186/s13073-023-01256-6

**Published:** 2023-12-01

**Authors:** Chen Tang, Shaliu Fu, Xuan Jin, Wannian Li, Feiyang Xing, Bin Duan, Xiaojie Cheng, Xiaohan Chen, Shuguang Wang, Chenyu Zhu, Gaoyang Li, Guohui Chuai, Yayi He, Ping Wang, Qi Liu

**Affiliations:** 1 Key Laboratory of Spine and Spinal Cord Injury Repair and Regeneration (Tongji University), Ministry of Education, Orthopaedic Department of Tongji Hospital, Frontier Science Center for Stem Cell Research, Bioinformatics Department, School of Life Sciences and Technology, Tongji University, Shanghai, China; 2 Translational Medical Center for Stem Cell Therapy and Institute for Regenerative Medicine, Shanghai East Hospital, Frontier Science Center for Stem Cell Research, Bioinformatics Department, School of Life Sciences and Technology, Tongji University, Shanghai, 200092 China; 3 Department of Medical Oncology, Shanghai Pulmonary Hospital, Tongji University Medical School Cancer Institute, Tongji University School of Medicine, Shanghai, 200433 China; 4 Tongji University Cancer Center, Shanghai Tenth People’s Hospital of Tongji University, Tongji University, Shanghai, China; 5 Research Institute of Intelligent Computing, Zhejiang Lab, Hangzhou, 311121 China; 6 Shanghai Research Institute for Intelligent Autonomous Systems, Shanghai, 201210 China

**Keywords:** Single-cell RNA-seq, Immunotherapy, Combination therapy optimization, Bipartite graph, Computational pipeline, Web server, Precision medicine

## Abstract

**Background:**

The precise characterization of individual tumors and immune microenvironments using transcriptome sequencing has provided a great opportunity for successful personalized cancer treatment. However, the cancer treatment response is often characterized by in vitro assays or bulk transcriptomes that neglect the heterogeneity of malignant tumors in vivo and the immune microenvironment, motivating the need to use single-cell transcriptomes for personalized cancer treatment.

**Methods:**

Here, we present comboSC, a computational proof-of-concept study to explore the feasibility of personalized cancer combination therapy optimization using single-cell transcriptomes. ComboSC provides a workable solution to stratify individual patient samples based on quantitative evaluation of their personalized immune microenvironment with single-cell RNA sequencing and maximize the translational potential of in vitro cellular response to unify the identification of synergistic drug/small molecule combinations or small molecules that can be paired with immune checkpoint inhibitors to boost immunotherapy from a large collection of small molecules and drugs, and finally prioritize them for personalized clinical use based on bipartition graph optimization.

**Results:**

We apply comboSC to publicly available 119 single-cell transcriptome data from a comprehensive set of 119 tumor samples from 15 cancer types and validate the predicted drug combination with literature evidence, mining clinical trial data, perturbation of patient-derived cell line data, and finally in-vivo samples.

**Conclusions:**

Overall, comboSC provides a feasible and one-stop computational prototype and a proof-of-concept study to predict potential drug combinations for further experimental validation and clinical usage using the single-cell transcriptome, which will facilitate and accelerate personalized tumor treatment by reducing screening time from a large drug combination space and saving valuable treatment time for individual patients. A user-friendly web server of comboSC for both clinical and research users is available at www.combosc.top. The source code is also available on GitHub at https://github.com/bm2-lab/comboSC.

**Supplementary Information:**

The online version contains supplementary material available at 10.1186/s13073-023-01256-6.

## Background

Precision medicine is a promising concept and future trend in cancer therapy, focusing on matching the appropriate treatment for each cancer patient based on the individual omics profile of their tumor [1–3]. Recent studies have indicated that the application of personalized and precise treatment can substantially improve the survival of tumor patients, which has achieved increased attention in effectively boosting such prototype studies in the clinic [4–8]. Previous modeling of treatment response was mostly based on in vitro cell line assays or gene expression from bulk transcriptome sequencing of tumor samples by quantifying the average profile of the whole tumor in each patient [9–11]. However, cancer is an extremely heterogeneous disease that introduces variations not only between cancer cells from different patients but also between cancer cells within each patient [12, 13], and clinical predictions of patient responses to medications targeting tumor heterogeneity at a single cell level have not been fully elucidated. In addition, tumor and immune cell interactions, which characterize the tumor microenvironment, also have a great impact on cancer treatment. Such heterogeneity and complexity of individual tumors are associated with tumor progression [14] and drug resistance [15], which is challenging for precision medicine applications in clinical practice.

To overcome the challenges of intratumor heterogeneity, drug combination therapy is applied to discover optimal drug sets targeting different tumor and immune cell types by investigating marker genes and biological pathways. Several computational methods have been developed to identify the optimal drug combination using in vitro datasets [16–18] or bulk RNA sequencing (RNA-seq) [19–22], but they are still limited to being in vitro or limited to solving tumor heterogeneity for cell groups with divergent expression but quantified by the average in bulk sequencing. Single-cell RNA sequencing (scRNA-seq) technique provides the gene expression of cell types at the single-cell level and has been widely applied to uncover the high-resolution tumor microenvironment landscape in various cancer types [23–31], providing an opportunity to overcome the limitations of bulk RNA-seq methods in intratumor heterogeneity evaluation. Recently, a computational tool has been developed to optimize drug combinations with single-drug perturbation data using cell mass cytometry time-of-flight technique, revealing the advantage of single-cell technique in addressing tumor heterogeneity issues for drug combination optimization [32]. Several recent studies have also developed computational protocols and methodologies to identify drug combinations based on patient-derived cells or targeting tumor heterogeneity through single-cell sequencing [8, 18, 22, 30], suggesting a boosting power of using comprehensive single-cell-level heterogeneity datasets for drug combination optimization; however, further study is waiting to be fully conducted.

In addition, the revolutionary strategy of immunotherapy, focusing on immune checkpoint blockade (ICB) immunoregulatory pathways such as programmed cell death-1 (PD-1)/programmed death-ligand 1 (PD-L1) signaling axis, has achieved effective treatment responses in a portion of clinical cases [33–38]. Responses to checkpoint inhibitor immunotherapy are highly correlated with the levels of tumor immune infiltration and patient immune activity [39]. However, only 13% of patients satisfying the immunotherapy requirement of immune infiltration and immune activity can derive a long-term response to ICB monotherapy [40]. Moreover, the current transcriptome-based approach only estimates tumor-infiltrating immune cells for immunotherapy patients but neglects complex and heterogeneous tumor cells and their interactions with immune cells in the tumor microenvironment (TME). Combining small molecules and ICB to mediate TME and boost immunotherapy serves as a promising strategy for effective tumor treatment; however, how to rationally identify such small molecules from a large compound space that can be paired with ICB for effective combinational therapy based on the precise characterizing of the TME is challenging.

To explore all these issues in personalized tumor therapy, for the first time, we present a feasible precision oncology treatment prototype, comboSC (personalized tumor combination therapy optimization based on the single-cell transcriptome), to perform personalized cancer combination therapy optimization using scRNA-seq, which aims to maximize the translational potential of the in vitro cellular response for identifying synergistic drug combinations and prioritizing them for personalized clinical usage with single-cell RNA-seq. Specifically, comboSC first quantitatively characterizes the personalized tumor microenvironment of individual tumors using scRNA-seq data in vivo and then performs combination therapy optimization for both immunotherapy and targeted therapy based on transcriptomic profiles of both tumor and immune cell groups, taking advantage of the largely existing in vitro pharmacogenomics data from LINCS [11, 41], Connectivity Map (CMap) [10], and Genomics of Drug Sensitivity in Cancer (GDSC) [42, 43]. We applied comboSC to a comprehensive set of 119 tumor samples from 15 cancer types, including solid tumors and blood tumors, and indicated its generalized utility and advantages in combination therapy optimization across different cancer types. Overall, comboSC provides a feasible and one-stop computational framework and a proof-of-concept study to predict potential drug combinations for further experimental validation and clinical usage, which will facilitate and accelerate personalized tumor treatment by reducing screening time from a large drug combination space and saving valuable treatment time for individual patients. A user-friendly comboSC web server for both clinical and research users is available at www.combosc.top.

## Methods

### Data collection

We collected the single-cell RNA-seq (scRNA-seq) data of cancer patients as comprehensively as possible from literature mining and scRNA-seq databases [44, 45] (Additional file 2: Table S1). All scRNA-seq data used in this study were sequenced before the treatment of target therapy and immune therapy, including anti-PD1/PDL1 therapy and anti-CTLA4 therapy. Over 360,000 cells from 119 samples, 15 cancer types including solid tumors and blood tumors, and 2 sequencing platforms were analyzed by comboSC in this paper (Additional file 2: Table S2). Cancer cell line transcriptional profiles were obtained from the Cancer Cell Line Encyclopedia (CCLE) (https://portals.broadinstitute.org/ccle/home) [46, 47]. Pharmacogenomic data for CCLE datasets were downloaded from Genomics of Drug Sensitivity in Cancer (GDSC) [42, 43] and Connectivity Map (CMap) [10]. The pharmacogenomics data in GDSC and CMap were used to predict the drug response to the patient (Additional file 2: Tables S3-4). Drug-drug interactions were predicted by DrugComb [48]. We collected the recommended drug combinations in clinical studies from ClinicalTrials.gov.

### Single-cell RNA data preprocessing

To preprocess the scRNA-seq data, comboSC first uses the Cell Ranger toolkit (version 3.1.0) provided by 10 × Genomics to aggregate raw data, filter low-quality reads, align reads to the human reference genome (GRCh38), assign cell barcodes, and generate the unique molecular identifier (UMI) matrix. Then, comboSC uses Seurat v3 [49] to analyze the scRNA-seq data. Specifically, the raw UMI matrix is processed to filter out cells with fewer than 200 genes or greater than 5% mitochondrial gene counts to ensure that most of the heterogeneous cell types are included in downstream analyses. Next, for general applicability to highly variable personalized tumor scRNA-seq profiles, comboSC takes the following steps to reduce potential noise and artifacts. For scRNA-seq datasets with a high dropout rate, comboSC used SAVER [50] to correct abnormal read counts and recover the gene profiles by neighboring genes and cells. For the integrative analysis of large-scale scRNA-seq datasets from different experiments and different sequencing platforms, comboSC uses matching mutual nearest neighbors (MNN) from Seurat v3 [49] to remove potential batch effects. Last, comboSC performs global cell clustering and cell annotation for immune-related cell types as tumor-related cell types in each sample by marker genes from the CellMarker database [51]. ComboSC applies copycat [52] to annotate malignant cells by copy number variation. For TISCH datasets [44, 45], we retrieved the raw scRNA expression count files and then applied them to the comboSC data preprocessing step, which was consistent with the data preprocessing step for other scRNA-seq samples in our study. Specifically, we used the well-curated cell annotations from the TISCH database, where manual cell annotation is recommended to use manual cell annotations in the comboSC preprocessing step. For drug combination prediction, we discarded two datasets with too few malignant cells (< 100) or too few T cells (< 60).

### Personalized immune score calculation by the Tres model

**Fig. 1:**
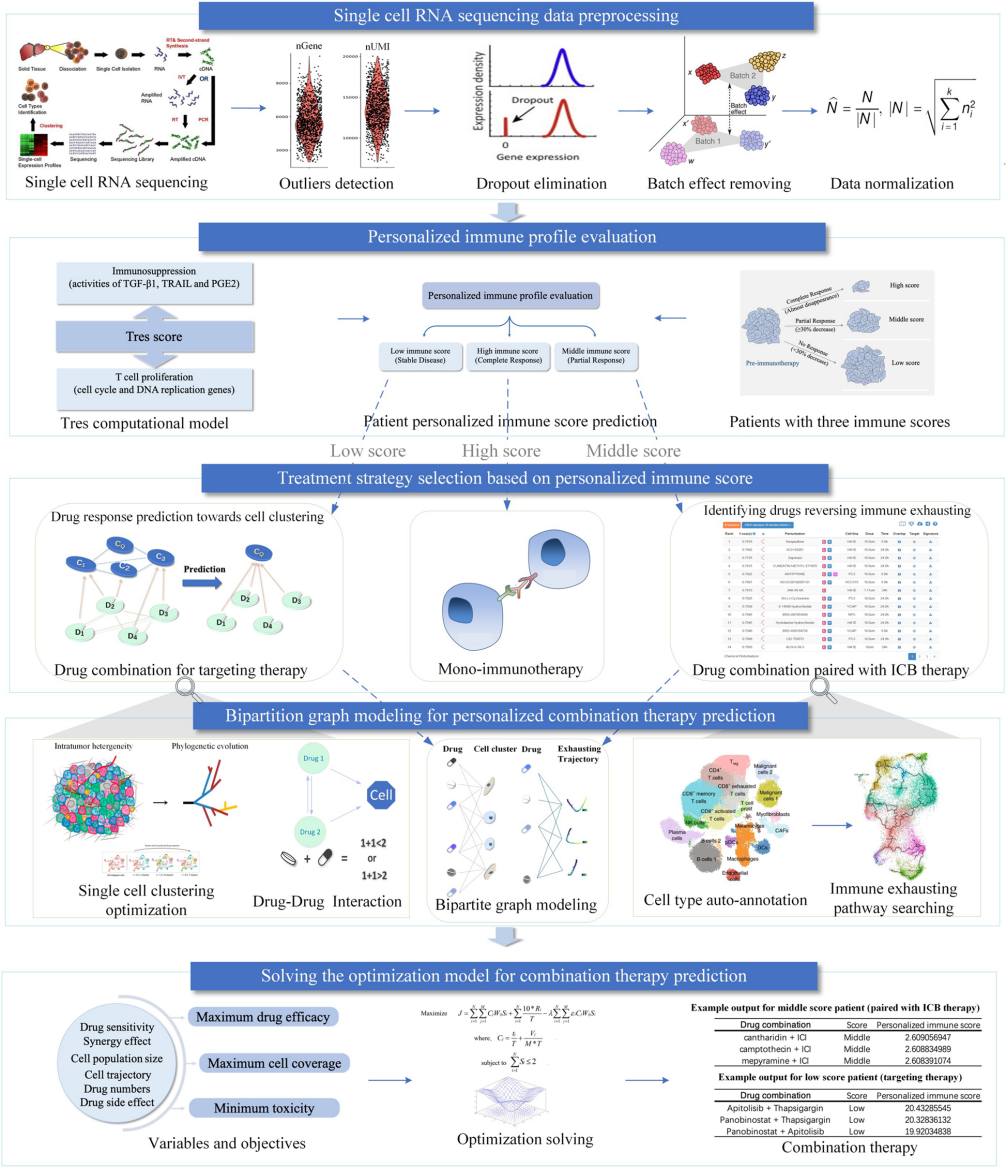
ComboSC precision medicine framework. comboSC comprises five modules: **a** Single-cell RNA sequencing data preprocessing. **b** Personalized immune profile evaluation. **c** Treatment strategy selection based on immune score. **d** Bipartition graph modeling for personalized combination therapy prediction. **e** Solving the optimization model for optimal combination therapy prediction

ComboSC uses the T cell resilience (Tres) model [39] for calculating immune scores to predict personalized immunotherapy response levels (Fig. 1b). Tres model utilizes single-cell and bulk RNA-seq data to capture signatures of T cell group resilient to immunosuppressive signals and successfully predicts clinical responses for patients who received immune-checkpoint inhibitors immunotherapy for melanoma, lung cancer, triple-negative breast cancer, and B cell malignancies [39]. Specifically, the Tres model constructs a multivariate linear regression by three groups of variables, including cytokine signaling activities, T cell proliferation signatures, and interactions between the above two variables. The cytokine signaling activities indicate the personalized immunosuppression level, which is calculated by CytoSig [53] with TGF-β1, TRAIL, and PGE2 signaling, and the T cell proliferation signatures are calculated by modeling from all genes in cell cycle and DNA replication pathways, as well as genes for T cell cytotoxicity through a linear regression approach.

ComboSC first identified all T cell subgroups from the input scRNA-seq dataset, including CD4 T cells and CD8 T cell subgroups [39]. Following the methods from the Tres model paper [39], comboSC next calculated the aggregated scRNA profile for all T cells in each sample as a pseudo-bulk profile and normalized the gene expression using ${\mathrm{log}}_{2}\left(\frac{\mathrm{TPM}}{10}+1\right)$. The scRNA-seq gene expression was further scaled to zero mean across all input samples. Consistent with the Tres model [39], comboSC used the score of 0 as the cutoff for responsive samples with high or middle immune scores and non-responsive samples with low immune scores, and the complete response samples were defined as the top 10% samples among all input samples [40], as other samples with score over 0 but lower than the high immune score samples were defined as middle immune score samples. We used 63 samples to evaluate the performance of the Tres model score for immunotherapy response. The ROC curve and the multi-class AUROC value were calculated using the pROC package in R. The personalized response to immune checkpoint blockade therapy was measured using Response Evaluation Criteria in Solid Tumors (RECIST) v1.1. We applied the same classification criteria of complete responders (CR) and partial responders (PR) as those of RECIST and took patients of stable disease (SD) and progressive disease (PD) as non-responders (NR).

### Inferring drug response for single-cell RNA-seq cell clusters

To infer the drug response to specific malignant cell clusters, we collected the cancer cell line bulk RNA-seq data from the CCLE database [46, 47] and corresponding drug response annotations for each cell line from the GDSC database [42, 43]. Similar to previous literature [54, 55] projecting bulk RNA-sea data to single-cell RNA-seq data, comboSC takes a similarity-based calibration method scmap [56], which calculates the similarity of gene expression profiles between malignant cell groups in individual tumors and CCLE cell lines and assigns drug responses to cell groups by drug responses from CCLE cell lines with high similarity. Briefly, scmap first represents each cell cluster by the median expression of each gene and then calculates the similarity of cell clusters to the query reference as the CCLE cell line gene expression, which also uses unsupervised feature selection to include only the genes that are most relevant for the underlying biological differences and overcomes the batch effect. This projection method is applied in comboSC for both cell clustering and drug response prediction.

### Drug response-driven malignant cell Louvain clustering

To identify heterogeneous malignant subgroups from all malignant cells, comboSC takes a drug response-driven Louvain clustering method to group cells with similar drug responses in the same cell cluster and achieve stable subclusters for single-cell analysis [49]. The basic idea of such drug response-driven Louvain clustering is to set the resolution parameter *r* in routine Louvain clustering automatically, which will dynamically determine the cluster number. Specially, the “FindClusters” function in Seurat contains a resolution parameter *r* that sets the “granularity” of the downstream clustering, with increased values leading to a greater number of clusters, while this parameter is set manually in the traditional study. In our study, comboSC maximizes the following objection function* J* to represent the drug response specificity in each cell cluster and maximizes *J* to optimize the clustering resolution parameter *r* automatically using the following functions:

1 $$\mathrm{Maximize\,}J\left(r\right)=\,\frac{M\left(r\right)}{10}+\frac{\sum \mathrm{S}\left(i\right)*\mathrm{cell}\_\mathrm{number}\left(\mathrm{i}\right)}{T}$$

2 $$\mathrm{Wh}\mathrm e\mathrm r\mathrm e\,S\left(i\right)=\left\{\begin{array}{l}\begin{array}{cc}1,&\,\mathrm{if}\;\mathrm{sim}\left(i\right)>\alpha\end{array}\\\begin{array}{cc}0,&\mathrm{if}\;\mathrm{sim}\left(i\right)\,\leq\alpha\end{array}\end{array}\right.$$

subject to:

3 $$\frac{T}{\mathrm{10,000}} <r<\frac{T}{2000}$$

4 $$\sum_{k=1}^{N}\sum_{i=2}^{M\left(r\right)}\sum_{j=1}^{i-1}{\left({W}_{ki}\left(r\right)-{W}_{kj}\left(r\right)\right)}^{2}>\varepsilon$$

Here, *r* represents the resolution used in Louvain clustering, and $M\left(r\right)$ represents the number of cell clusters under resolution* r*. *i* and* j* represent the *i*th and *j*th cell cluster, respectively. The coefficient 1/10 for $M\left(r\right)$ is used to adjust the range of the first item similar to the second item, ranging from 0 to 1. *k* represents the *k*th drug, and *N* represents the total number of drug candidates. cell_number(*i*) represents the number of cells in the *i*th cell cluster, and *T* represents the total number of malignant cells. The coefficient of *T* is determined as 1/10,000 and 1/2000 since the resolution *r* would be limited to ranges from 0.3 to 1.5 for *T* of 3000 cells, which is less restricted than Seurat “FindClusters” function, which limits the resolution *r* from 0.6 to 1.2. sim(*i*) represents the similarity between the *i*th cell cluster and the reference CCLE cell lines, calculated as the maximum similarity between each cell in the cluster and each CCLE cell line. *W*_*ki*_(*r*) and *W*_*kj*_(*r*) represent the response of the *k*th drug for the *i*th and *j*th cell clusters, respectively, with the resolution* r*. The intuition of Eq. 1 is to maximize the resolution as well as the number of responsive cells among the clusters that are similar to known CCLE cell lines, and the similarity threshold is set as $\alpha$ in Eq. 2. The intuition of Eq. 4 is to set a threshold $\varepsilon$ to constrain the similarity between any two responses given the *i*th cluster and *j*th cluster and the *k*th drug, so as to avoid an infinite increase in resolution. In this study, $\alpha$ is set to 0.4 (Additional file 1: Fig. S1), and $\varepsilon$ is set to 1.

### Cell type-specific drug response prediction for low immune score samples

ComboSC utilizes tumor cell expression data from the CCLE database [46, 47] and corresponding drug response data for CCLE expression data from GDSC [42, 43] for cell type-specific drug response prediction. Based on the highest similarity score of the CCLE cell line to each cell group, comboSC uses different drug response assignment strategies. When the highest similarity score is above 0.8, comboSC directly assigns the drug response of the best-matched CCLE cell line to the cell group. If the highest similarity score is above 0.4 but below 0.8, which indicates a partial correlation between the cell group and CCLE cell lines, comboSC applies the deep learning method CaDRReS [57] to refine similarity from the top similar CCLE cell lines and assign an adjusted drug response to the cell group. ComboSC would ignore the distinct cell group if all CCLE cell lines showed a low similarity score (< 0.4) to the cell group (Additional file 1: Fig. S1).

### Immune exhaustion-based drug response prediction for middle immune score samples

ComboSC predicts drug response to inhibition or reversal of immune cell exhaustion from immune exhaustion-related trajectories. First, the trajectory inference of the tumor immune microenvironment is applied to scRNA-seq data with Monocle3 (https://cole-trapnell-lab.github.io/monocle3) [58, 59], since the immune microenvironment is characterized by tumor cell clusters and immune cell clusters in each sample from the comboSC pre-processing step. ComboSC focuses on four ubiquitous immune-exhausting trajectories related to the modulation of the antitumor immune response, including (1) active T cell exhaustion, (2) memory T cell exhaustion [46], (3) tumor-associated macrophage differentiation, and (4) cancer-associated fibroblast (CAF) differentiation, and defines the trajectory start cell type and end cell type for each trajectory from the previous knowledge of these trajectories. Second, differentially expressed gene (DEG) analysis is applied to each immune-exhausting trajectory to identify trajectory signature genes if the trajectory existed in the input scRNA-seq dataset. If the trajectory exists, the trajectory score is computed by the cell count of the trajectory end cell type divided by the cell number of the trajectory start cell type. Also, if the cell types of the cell trajectory did not exist in the single-cell dataset, comboSC took “NA” as the trajectory score to the next step. Finally, comboSC predicts the score of the small molecular drug response to the signature genes of the immune exhaustion-related trajectory by L1000CDS [41], a pharmacogenetic search engine that calculates the cosine similarity of the small molecular drug response to cell groups by the overlap between the input DEGs and the drug response signature genes from CMap [10] and enables users to find small molecule signatures that match query gene expression signatures. ComboSC calculates the input DEGs by contrasting the cell groups at the start and end of the selected cell trajectory using Seurat [49] and then searches the DEGs in the L1000CDS [41] tool (Application Programming Interface) API (https://lincsproject.org/LINCS/tools/workflows/search-lincs-metadata-through-apis) for drug response predictions.

### Predicting small molecule/drug combination therapy response with a bipartite graph network

For patients with middle or low immune scores, comboSC applies a bipartite graph network to model the relationship between drug responses and personalize scRNA-seq profiles from patients (Fig. 1). Bipartite graph network model is able to provide a maximum coverage of the diverse molecular cell phenotypes of tumor and immune cell groups with parsimonious drug combinations considering various information and constrains, including cell population size, cell trajectory, drug synergy effect, drug side effect, and drug toxicity. First, comboSC takes the targeted cell cluster or developmental trajectory as the left nodes of the bipartite graph network, and a larger cell population size would be assigned a higher weight. ComboSC integrates the static profile for each cell (obtained from scRNA-seq) and the dynamics of gene expression with the estimated RNA velocity (calculated by velocyto [60]) for each cell cluster. The latter predicts the fate of each single cell by its ratio of unspliced and spliced mRNA to mature mRNA from the read mapping file, and such possible future cell fate trends will also guide the prediction of drug combinations. Second, comboSC takes cancer drugs as the right nodes of the network. Finally, comboSC considers both positive drug response and side effects, including antagonism and drug toxicity to the cell cluster as the edges between nodes from both sides. Specifically, comboSC uses drug response annotations from the GDSC database [42, 43] for cell line datasets in the CCLE database^68^ and drug toxicity from the SIDER database [61]. The edges in the comboSC bipartite graph network represent the relationship between the multimodal views of the selected cells and the comprehensive effects of the drugs described above. Given the complexity of multiple object solutions for the bipartite graph network, comboSC applies a multi-objective programming function to solve the model and identifies the optimal drug combinations with the score *J* as follows:

5 $$\mathrm{Maximize}\,J=\sum_{i=1}^{N}\sum_{j=1}^{M}{C}_{j}{W}_{ij}{S}_{i} + \sum_{i=1}^{N}\frac{10*{R}_{i}}{T}-\lambda \sum_{i=1}^{N}\sum_{j=1}^{M}{\varepsilon }_{i}{C}_{j}{W}_{ij}{S}_{i}$$

6 $$\mathrm{where},\,{C}_{j}=\,\frac{{t}_{j}}{T}+\frac{{V}_{j}}{M*T}$$

7 $$\mathrm{subject\,to}\,\sum_{i=1}^{N}{S}_{i}\le\,2$$

where $J$ represents the goal of the optimization function. $N$ represents the number of drugs in the database, $M$ represents the number of cell clusters, and $T$ represents the total number of cells. ${C}_{j}$ represents the weight of the *j*th cluster, which is determined by the current cell number in the *j*th cell cluster as ${t}_{j}$, the number of cells developing towards the *i*th cell cluster in RNA velocity analysis as ${V}_{j}$, and the number of cell clusters *M* (see Eq. 6). Here, for the *j*th cell cluster, for example, positive ${V}_{j}$ indicates the potential to increase the cell group size and increase the weight of the cell cluster ${C}_{j}$ by the second term of formula 6. A larger absolute value of ${V}_{j}$ indicates a higher impact of RNA velocity to the *j*th cell cluster. ${W}_{ij}$ is the sensitivity score of the *i*th drug to the *j*th cell cluster, which is calculated for the low and middle immune score samples, respectively, as described aforementioned. *S*_*i*_ represents the selection of the *i*th drug for combination therapy, with 1 or 0, corresponding to whether or not drug *i* is selected, and the maximum number of drug candidates in a combination is set to 2 as constrained by inequality 7. ${R}_{i}$ represents the number of cells responding to the *i*th drug, and *T* represents the total number of cells, indicating that the selected drug candidates should maximize the coverage of the responsive cells among all cells. ${\varepsilon }_{i}$ represents the drug side effect and drug toxicity score of the *i*th drug, and the score is 1 if the drug is reported with more than ten high frequency (> 30%) side effects from the SIDER database [61], and 0 vice versa. $\lambda$ is the penalty term for the drug side effect score, which is defined as 0.1 in this study.

### Drug combination response evaluation in head and neck patient-derived cell lines

We collected scRNA-seq data from three head and neck patient-derived cell lines (PDC) samples treated with five drug combinations at two different dosages (GSE117872) [62]. Each PDC scRNA-seq data was derived from a single patient and five drug combinations were used independently for PDC samples. The pseudo-bulk RNA-seq dataset used in comboSC-bulk and CMAP method was generated by aggregation of gene expression in all cells from scRNA-seq data with Seurat [49]. Specifically, comboSC used single-cell profiles and cell clusters to predict and rank drug combination responses for each patient cell line. ComboSC-bulk method used aggregated gene expression from all cells to predict and rank combination drug response by comboSC, which will not use cluster-specific gene profiles. The CMAP method, which took DEGs as input to the CMap database for drug response ranking, also used aggregated gene expression for all cells, and then identified differential expressed genes to corresponding normal samples as input. Since the CMAP method only predicts a single drug response from L1000CDS [41], drug combinations are ranked by the average rank of two drugs in CMAP predictions. We evaluated the relative ranking of drug combination responses from comboSC, CMAP, and comboSC-bulk by Discounted Cumulative Gain (DCG) [63, 64]. The DCG score is calculated as follows:

8 $$\mathrm{DCG}=\,\sum_{i=1}^{k}\frac{{r}_{i}}{{\mathrm{log}}_{2}\left(i+1\right)}$$

where *k* is the number of the input drug combinations, and ${r}_{i}$ is the relative ranking difference between comboSC predictions and experimental validations, calculated as $r=|{\mathrm{rank}}_{\mathrm{predict}}-{\mathrm{rank}}_{\mathrm{experiment}}|$ for each ${r}_{i}$. The lower DCG score means a higher ranking of drug combination response.

### Evaluating the performance of comboSC with the ClinicalTrials.gov database

To further evaluate the drug response predictions from comboSC, we searched comboSC drug combination predictions in all drug combinations registered on ClinicalTrial.gov. To search the candidate drug combinations, the keywords “drug1,” “drug2,” and “combined with” using the ClinicalTrial.gov API (https://clinicaltrials.gov/api/gui). For drug synonyms, the ClinicalTrial.gov API will auto-detect synonyms and return records for all drug synonyms. These drug combination records were further filtered with “phase II clinical trials completed,” which excluded drug combinations in which a phase I trial failed due to toxicity. Then, we estimated the ranking performance of comboSC drug combinations by the enrichment significance of the top 10% predictions registered in ClinicalTrials.gov, formulated as follows:

9 $$p=1-\sum_{i=0}^{k-1}\frac{\begin{pmatrix}M\\i\end{pmatrix}\ast\begin{pmatrix}N&-&M\\n&-&i\end{pmatrix}}{\begin{pmatrix}N\\n\end{pmatrix}}$$

where *N* is the number of all comboSC drug predictions, *M* is 10% of *N*, *n* is the number of drug combinations registered on ClinicalTrials.gov, and *k* is the number of the top 10% drug combinations registered on ClinicalTrials.gov.

### Using comboSC with an interactive web interface

The comboSC web server provides user-friendly interactive access for both clinical and research users. It was developed with a framework of Bootstrap, Vue, jQuery, and D3js. For online users, comboSC will take the scRNA expression matrix and the corresponding cell metadata matrix (the same cells in the expression matrix) as input, and then the web server will run the entire pipeline task in the background and automatically send an email containing the comboSC standard output, i.e., a data matrix of the top-ranked combination therapy results with detailed annotations when the task is completed.

## Results

### Overview of comboSC framework

ComboSC is a computational prototype for tumor combination therapy selection and response prediction based on personalized scRNA-seq data at a pan-cancer level. With the input of scRNA-seq profiles from individual tumor samples, comboSC provides the optimal personalized combination therapy with a response score after a series of calculations. comboSC mainly consists of five modules: (A) scRNA-seq data preprocessing, (B) personalized immune profile evaluation, (C) immune score-based treatment strategy selection, (D) bipartite graph modeling for combinatorial drug response prediction, and (E) modeling with a multi-objective optimization strategy (Fig. 1). The following are short descriptions of comboSC pipeline:

#### Step 1: scRNA-seq data preprocessing

**Fig. 2:**
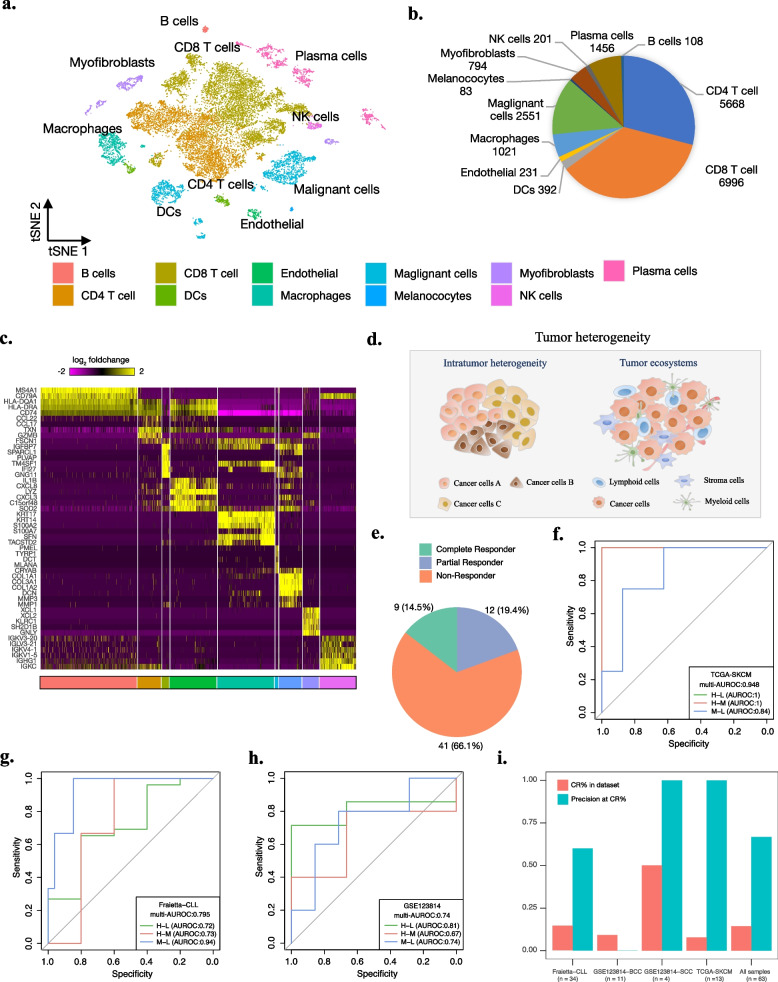
Personalized immune profile evaluation. **a** The t-SNE plot shows the immune microenvironment of patients from single-cell transcriptome sequencing data using the comboSC workflow. The different colors represent the cell types annotated by comboSC. **b** The pie chart shows the proportion of cell groups in the tumor ecosystem and the population size of each cell group. **c** The heatmap shows the marker genes and their expression patterns in identified immune cells, malignant cells, and stromal cells. **d** A schematic description of tumor heterogeneity used in comboSC. **e** The proportion of CR, PR, and NR samples in 63 samples. **f**–**h** ROC curves of comboSC immune score from Tres model in **f** TCGA-SKCM, **g** Fraietta-CLL, and **h** GSE123814 datasets. H, M, and L in the figure indicate CR, PR, and NR samples, respectively. ROC curves and AUROC values for H–L, H-M, and M-L indicate the classification performance of CR versus NR, CR versus PR, and PR versus NR using comboSC immune scores, respectively. **i** The bar plot shows the precision of CR in top immune score samples for each dataset in the green left bar. The red right bar shows the threshold of top immune score samples in each dataset, which is defined as the proportion of CR samples

Due to the complexity and diversity of input datasets from different sequencing platforms [56], comboSC first performs a series of data preprocessing procedures, including outlier detection, dropout elimination, batch effect removal, and data normalization (see the “Methods” section). Next, comboSC performs automated global cell clustering and annotation for immune cells, malignant cells, and stromal cells and then calculates the population size and expression levels of each cell group, which enables the recognition of sample heterogeneity and identification of cell types related to tumor immune microenvironment evaluation in downstream sections (Fig. 2a–d).

#### Step 2: Personalized immune profile evaluation

Learning from a previous study for clinical immunotherapy response prediction, comboSC first uses the signature genes of tumor-resilient T cells from scRNA-seq to calculate the immune score by the T cell resilience (Tres) model^39^. The immune score is then determined as high, middle, or low by certain threshold of the Tres score, representing complete response, partial response, or no response for immunotherapy (see the “Methods” section).

#### Step 3: Treatment strategy selection based on personalized immune score

For high immune score samples, comboSC recommends routine immunotherapy strategies such as immune checkpoint inhibitor treatment since the immune function of the sample is properly activated. For middle immune score samples, inspired by recent findings of the immunostimulatory potential of chemotherapy and targeted therapy [65, 66], comboSC recommends a combination strategy with certain small molecules/drugs to enhance the potential response to immunotherapy. For low immune score samples that show a low potential response to immunotherapy, comboSC recommends the small molecule/drug combination therapy strategy to eliminate malignant cells directly.

#### Step 4: Bipartite graph modeling for combinatorial drug response prediction

Bipartite graph is a graph representation of two disjoint sets, like drugs and cells with edges only connecting nodes from different sets, as the relationship between drug and cell can be learned by graph modeling. The bipartite graph model has been successfully applied for discovering the gene and drug combinatorial effects [67, 68]. For individual samples with both middle and low immune scores, comboSC uses the bipartite graph network to infer the relationship between candidate drugs and different cell clusters. The basic idea of the bipartite graph network model applied here is to provide a maximum coverage of the diverse molecular cell phenotypes (both tumor and immune cell groups) with parsimonious drug combinations considering various aspects including cell population size, cell trajectory, drug synergy effect, drug side effect, drug toxicity, etc., rather than simply providing single drug or drug combinations effectively responded among the top majority tumor cell groups [69]. For low immune score samples, comboSC infers the individual drug response and drug-drug interactions to the levels of malignant cell subclusters from the bipartite graph network (see the “Methods” section). Similarly, for middle immune score samples, comboSC uses bipartite graph network modeling to infer the drug response to immune cell dynamics obtained from differential genes of cell trajectories associated with immune cell exhaustion (see the “Methods” section).

#### Step 5: Solving the model with a multi-objective optimization strategy

Taking advantage of the bipartite graph model, comboSC comprehensively integrates both treatment response and side effects such as antagonism and drug toxicity of drug combinations into the single-cell profile by considering all target immune cell clusters or malignant cell clusters. The final optimization problem in comboSC is tractable and quadratic in the number of drugs because all other factors are combined into a single heuristic objective function and the maximum number of drugs considered is two.

### ComboSC predicts immunotherapy response by the Tres model

To evaluate the tumor immune profile and immunotherapy response prediction of comboSC, we applied comboSC to a comprehensive set of 119 tumor samples of 15 cancer types, including basal cell carcinoma (BCC), breast invasive carcinoma (BRCA), colorectal cancer (CRC), head and neck cancer (HNSC), uterine corpus endometrioid carcinoma (UCEC), non-small cell lung cancer (NSCLC), pancreatic adenocarcinoma (PAAD), skin cutaneous melanoma (SKCM), liver hepatocellular carcinoma (LIHC), adult acute myeloid leukemia (AML), uveal melanoma (UVM), thyroid carcinoma (THCA), kidney renal clear cell carcinoma (KIRC), synovial sarcoma (SS), and osteosarcoma (OS). As shown in the workflow in Fig. 1, we first performed a global cell clustering and identified T cells from the tumor microenvironment. Next, we calculated the immune scores of these samples generated by comboSC using the Tres model [39], an accurate immunotherapy response prediction model for both bulk RNA-seq and single-cell RNA-seq by modeling both immunosuppressive signals and proliferation signals in T cells (see the “Methods” section). To estimate the consistency of immune score to the personalized immunotherapy response, we evaluated the immune scores using three collected datasets of 62 samples with known immunotherapy response, including one scRNA-seq dataset (GSE123814) with 11 BCC samples and 4 squamous cell carcinoma (SCC) samples, and 2 bulk RNA-seq datasets, including 34 chronic lymphocytic leukemia (CLL) samples from Fraietta et al. [70] and 13 SKCM samples from TCGA data portal [71]. Overall, 9 complete response (CR) samples, 12 partial response (PR) samples, and 41 no response (NR) samples were collected (Fig. 2e, Additional file 2: Table S5). We used the multiclass AUROC value to evaluate the performance of comboSC immune scores for three immunotherapy response groups in each dataset. Overall, comboSC immune scores showed high accuracy in three datasets, with corresponding AUROC values of 0.795, 0.948, and 0.74 in three datasets, respectively (Fig. 2f–h), and 0.814 overall. Specifically, we further calculated the accuracy of comboSC immune score for the CR group, which was not evaluated in the original Tres model study. Since the proportion of CR samples varies between datasets, we used the fraction of CR samples in each dataset as the threshold and showed a precision of 0.6 (3/5), 1 (1/1), and 1 (2/2) in CLL, SKCM, and SCC datasets, respectively, but did not identify CR in BCC dataset (0/1), which showed the precision of 0.667 (6/7) in all evaluated datasets and revealed a high precision of comboSC immune score for complete response sample classification (Fig. 2i). Overall, the Tres model can be taken as an acceptable model for patient stratification in terms of single-cell RNA-seq with comprehensive validation support. Therefore, finally, we applied the Tres model to 119 tumor single-cell RNA-seq datasets and identified 20 high, 44 middle, and 55 low immune score samples.

### ComboSC provides accurate response predictions of small molecule drug combination therapy for samples with low immune scores

In this study, we performed a comprehensive analysis to show the utility of comboSC for 55 samples with low immune scores. Small molecule drug combinations are recommended as these samples are demonstrated to have a limited immune response.

**Fig. 3:**
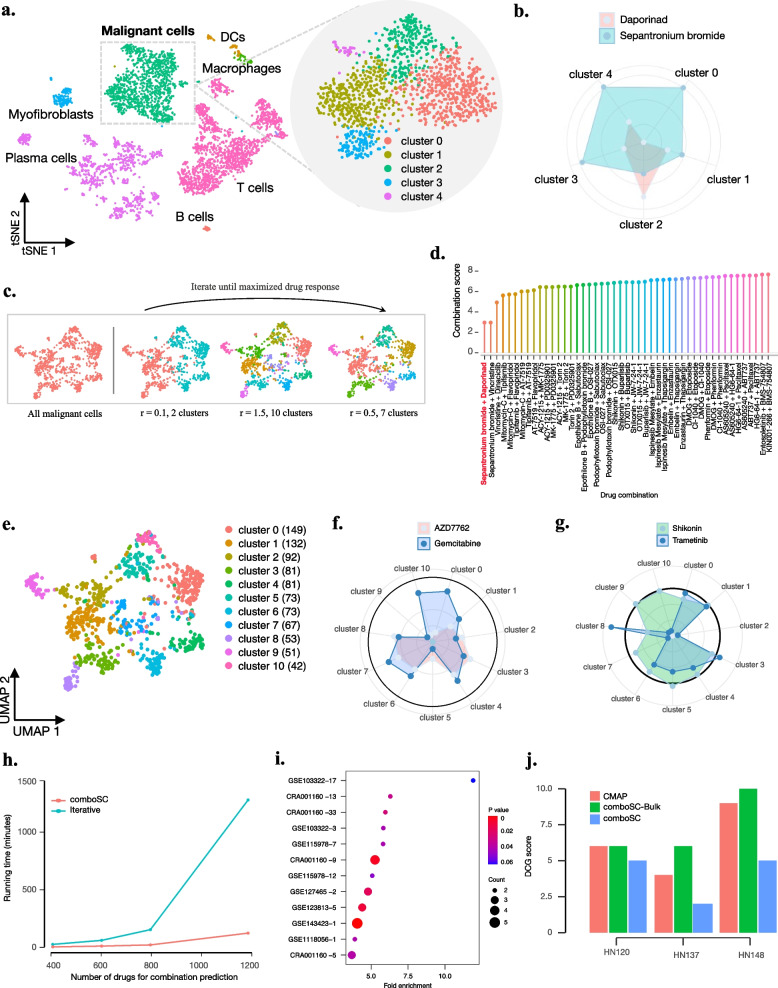
ComboSC provides accurate response predictions for small-molecule drug combination therapy. **a** t-SNE plot for immune cells and malignant cells in sample su006 (GSM3511756). The colors on the left represent the different cell groups annotated by comboSC. The circled malignant cells in the left t-SNE plot are further divided into five subclusters in the right circle according to their various drug responses. **b** Radar map showing distinct drug responses of daporinad and sepantronium bromide in five malignant cell subclusters. **c** Overview of drug response-driven malignant cell graph clustering. ComboSC repeats the iteration for different graph cluster resolution until the drug response is maximized. **d** Top-ranked drug combinations in comboSC predictions for su006. The height of the bars represents the drug combination score. **e** UMAP plot for eleven malignant cell clusters in a breast cancer sample (GSE176078). **f** Radar map showing distinct drug responses of transfer learning-based method for the top two drugs AZD7762 and gemcitabine in eleven malignant cell subclusters. The drug response score at the black highlight circle is 12, equal to the black highlight circle in **g**. **g** Radar map showing the distinct drug responses of comboSC for the top drug combination shikonin and trametinib in eleven malignant cell subclusters. The drug response score at the black highlight circle is 12, equal to the black highlight circle in **f**. **h** Comparison of calculation time under different drug numbers between comboSC optimization and the traditional iterative method. **i** This bubble chart shows the count and enrichment significance of the top 10% of comboSC predictions registered in the ClinicalTrials.gov database. The *x*-axis is the fold enrichment of clinically validated drugs in the top 10% of comboSC predictions compared to all comboSC predictions, and the *y*-axis is the dataset. The color represents the *p* value of the enrichment analysis. **j** Performance of comboSC, comboSC-bulk, and CMAP predictions in three PDC datasets. The *y*-axis is the DCG score, with a lower DCG score indicating higher ranking accuracy

We started by applying comboSC to a BCC single-cell RNA-seq dataset before anti-PD1 immunotherapy (GSM3511756) [72] as a demonstration example to show the underlying processing logic of comboSC. These samples are assigned a low immune score by comboSC, indicating that it is suitable to use small molecule drug combinations for personalized therapy. After the data preprocessing procedure, a global landscape was automatically drawn by comboSC, indicating the proportions of malignant cell populations and tumor microenvironment in the sample (Fig. 3a). ComboSC performed a drug response-driven graph clustering method on malignant cells (see the “Methods” section), which optimized cell clusters by grouping the cells of similar drug response into the same cell cluster. The drug response-driven graph clustering method is robust to variable graph clustering parameters and identifies five heterogeneous malignant cell subclusters with different drug sensitivities to different drugs (Fig. 3a–c). Specifically, sepantronium bromide, a small molecule proapoptotic agent with potential antineoplastic activity to deplete energy reserves in metabolically active tumor cells and induce tumor cell apoptosis, showed high drug sensitivity in the majority of the cell clusters and most of all cells (1179 cells out of a total of 1379 cells) but lower sensitivity than daporinad, another small molecule with potential antineoplastic and antiangiogenic activities to induce extrinsic or intrinsic apoptotic pathways and tumor cell apoptosis, in cell cluster 2 (218/1397) (Fig. 3b). Using the bipartite graph model, comboSC predicted optimal drug combinations for the BCC sample by jointly considering the malignant cell markers and synergistic drug combination candidates. Finally, comboSC identified the combination of sepantronium bromide and daporinad as the top prediction (Fig. 3d). Rather than simply providing a single drug or drug combination for an effective response among the top two tumor cell groups that account for the majority of tumor cells [69], comboSC identified drug combinations in response to most tumor cell groups with different molecular phenotypes.

Of note, recently, Daniel and his collaborators recently reported a single-cell RNA-seq-based transfer learning method to optimize drug combinations for BRCA samples [69], which aims to find a drug or a combination that is most effective among the top two cell lines representing the majority of tumor cells. To compare with this transfer learning-based method, we applied comboSC to a single-cell BRCA dataset [73] analyzed in their study using the same drug candidate combinations [16]. We first identified eleven tumor subclusters by the drug response-driven clustering (Fig. 3e). Then, we compared the drug response in target cell clusters for top drug combinations identified by the transfer learning-based method and comboSC. The top two drugs identified by the transfer learning-based method, AZD7762 and gemcitabine, showed similar responses in seven subclusters (646/894), and the corresponding drug combination did not show an advantage over the single top drug gemcitabine (Fig. 3f). In contrast, the comboSC top drug combinations identified by comboSC, shikonin and trametinib, showed a higher response in ten of eleven subclusters (802/894) than those of the transfer learning-based method, which indicated the advantage of predicting drug combinatorial effects with a higher resolution of tumor subgroups by comboSC (Fig. 3g).

Next, we applied comboSC to all 55 tumor samples to predict small molecule drug combination therapy, as these samples were assigned low immune scores and were predicted to have a limited immunotherapy response by comboSC. Taking advantage of bipartite graph representation along with multi-objective optimization, comboSC required less computational time and resources than the traditional computations: method for optimizing drug combination prediction (Fig. 3h). We further demonstrated the validity of the predicted drug combination by comboSC in the following three ways using literature evidence, clinical trial data, and gold standard drug combination perturbation data in patient-derived cell lines, and a detailed summary of these samples can be accessed in Additional file 2: Tables S2 and S5.

1. Among the prediction results, many known drug combinations with reported experimental validation evidence are ranked in the top predictions by comboSC from approximately 100,000 candidate drug combinations. These combinations served as an efficient therapeutic strategy with synergies, reduced toxicity, or prevention of tumor recurrence. For example, comboSC predicted a combination therapy of panobinostat and gemcitabine with a synergistic effect for NSCLC sample p4 in GSE127465 (ranked 5th). Gemcitabine is an antimetabolite that has demonstrated activity in the treatment of NSCLC, and numerous preclinical studies have demonstrated enhanced antitumor synergy when gemcitabine is combined with panobinostat [74]. For sample lbm2 in GSE143423, comboSC predicted the combination therapy of vinorelbine and gemcitabine with reduced toxicity (ranked 8th). Gemcitabine and vinorelbine have different mechanisms of antitumor activity, good therapeutic indices, and nonoverlapping toxicities. Their combination has shown promising results for the treatment of advanced NSCLC with fewer side effects [75]. For sample T7 in CRA001160, comboSC predicted gemcitabine combined with mitomycin-C therapy to prevent tumor recurrence (ranked 9th). It is interesting to see that many patients develop tumor recurrence under gemcitabine monotherapy, while the combination of gemcitabine and mitomycin-C could offer durable recurrence-free survival to tumor patients [76]. Overall, this literature evidence demonstrates the reliability of comboSC in terms of predicting drug combinations with synergy, reduced side effects, and tumor recurrence prevention.
2. For all 55 tumor samples, we evaluated drug combination predictions based on recommended drug combinations for distinct cancers from the ClinicalTrials.gov database. If the drug combinations predicted by comboSC from the GDSC and CMap databases [10] have been suggested to be effective or registered in clinical trials on ClinicalTrials.gov, this prediction will be considered relatively reliable. To this end, we performed an enrichment analysis of the predictions of comboSC, and it is encouraging to find that the top drug combinations from comboSC showed significant enrichment (*p* < 0.05) in drug combinations proven active in clinical trials, which indicates the consistency of the top-ranked drug combinations predicted by comboSC with that of the registered drug combinations in clinical trials (Fig. 3i).
3. To further investigate the performance of the ranking of drug combination predictions by comboSC, we compared the performance of comboSC, CMAP, and comboSC-bulk on a benchmark drug perturbation single-cell dataset of three patient-derived cell lines (PDC) derived from head and neck cancer patients (GSE117872) [62], where CMAP and comboSC-bulk took aggregated scRNA profiles as input. We used the rank of the response of the drug combinations to three PDCs measured from the previous study as the gold standard, including drug combinations of docetaxel/epothilone B, docetaxel/gefitinib, gefitinib/epothilone B, epothilone B/PI-103, and doxorubicin/vorinostat. As clinicians and researchers are more concerned or interested in drug combination predictions of higher ranks, we applied a metric of discounted cumulative gain (DCG), which represents the inconsistency between predicted rankings and actual rankings and gives higher weight to higher ranks, to evaluate the drug combination rankings for three benchmark methods, with lower DCG scores indicating higher accuracy in the ranking of drug combination predictions (see the “Methods” section). Of the three tumor cell lines, comboSC consistently showed lower DCG scores and higher accuracy than the other two methods (Fig. 3j), indicating the advantages of utilizing single-cell data for drug combination prediction in comboSC.

### ComboSC uncovers the potential of tumor immune microenvironment recovery from immune cell exhaustion dynamics for samples with middle immune scores

**Fig. 4:**
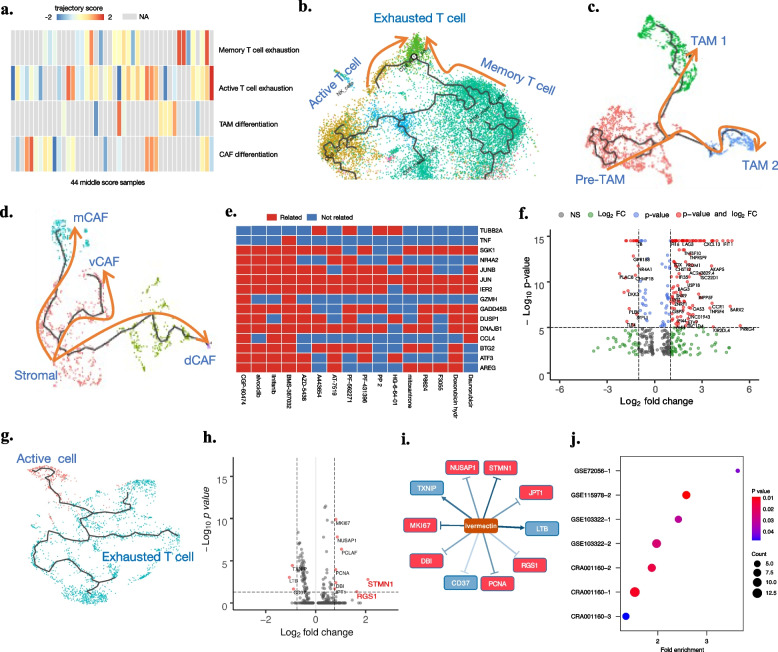
ComboSC uncovers the potential of tumor immune microenvironment recovery from immune cell exhaustion dynamics. **a** The heatmap shows trajectory scores of 44 middle immune score samples in four immune exhaustion cell trajectories. The gray color indicates that the trajectory does not exist in the sample. **b** Trajectory of active T cell exhaustion and memory T cell exhaustion. **c** Trajectory of tumor-associated macrophage (TAM) differentiation. **d** Trajectory of cancer-associated fibroblast (CAF) differentiation. **e** Drug-gene relationship used in comboSC, searched from the L1000CDS search engine. **f** The volcano plot identifies the differentially expressed genes along with the immune cell exhaustion trajectory in su008. Genes with *p* value < 10^−5^ and fold change > 2 are colored red. **g** Trajectory of T cell exhaustion in T15. **h** The volcano plot identifies the differentially expressed genes along with the immune cell exhaustion trajectory in T15. Genes with *p* value < 10^−5^ and fold change > 2 are colored red. **i** Drug effect of ivermectin to differentially expressed genes in Fig. 4h. The arrows and nocks between drug and genes represent the activation and suppression function of ivermectin to candidate genes, respectively. The red and blue colors of the gene labels represent upregulated and downregulated in the T cell exhaustion trajectory of T15, respectively. **j** This bubble chart shows the count and enrichment significance of the top 10% comboSC predictions registered in the ClinicalTrials.gov database. The *x*-axis is the fold enrichment of clinically validated drugs in the top 10% comboSC predictions compared to the ratio (60/1280) of validated drugs in all CMap drugs (Additional file 2: Table S4), and the *y*-axis is the dataset name and sample ID (Additional file 2: Table S2). The color represents the *p* value from the enrichment analysis, and the size represents the number of clinically validated drugs in the top 10% comboSC predictions

Of the patients who showed a treatment response to immunotherapy, only a fraction showed a complete response to immunotherapy alone, and most of the nonresponses and partial responses were caused by exhaustion of T cells [77]. Therefore, in this study, we further investigated the relative exhaustion levels of immune cells in 44 tumor samples with a middle immune score, where these samples have a partial immune response that has the potential to be enhanced. We identified significant differential immune exhaustion cell types and their cell trajectories in these middle immune score samples (Fig. 4a). Next, comboSC used a bipartite graph network to infer the relationship between drugs and immune cell trajectories and then predicted the drug combination response that can recover or enhance their immune status for immunotherapy. According to previous literature and top differential immune exhaustion cell types among samples, comboSC selected differentially expressed genes (DEGs) of four immune cell trajectories as drug targets to activate the immune response by immune cell recovery. These cell trajectories include (1) active T cell exhaustion, (2) memory T cell exhaustion [46], (3) direction of tumor-associated macrophage (TAM) differentiation [43], and (4) direction of cancer-associated fibroblast (CAF) differentiation (Fig. 4b–d). Interestingly, we identified one or more trajectories with different gene alteration levels in all 44 middle immune score samples (Fig. 4a), indicating that all these intermediate responsive samples have at least one trajectory to target to recover their immune status. Then, using the drug-gene relationships from the pharmacogenetic search engine L1000CDS [41] (Fig. 4e), comboSC predicted the candidate drug response to the dynamics of the selected trajectories for each sample.

Similarly, to evaluate the drug combination prediction by comboSC for middle immune score samples, we started by applying comboSC to identify the optimal drug combination for su008 of a BCC patient (GSE123814) as an example. First, we evaluated the immune profile of the su008 sample and found that the sample belonged to the middle immune level, indicating a partial response to immunotherapy. Combination therapy with small molecule drugs is required to improve the immunity level and treatment response to immunotherapy in this sample. Luckily, all four immune exhaustion trajectories above were identified in this sample, and the DEG analysis showed that the immune-related target gene of immunotherapy LAG3 [78] was altered along with the target cell trajectory (Fig. 4f). Gene Ontology (GO) analysis also showed that these DEGs were significantly enriched in immune-related functions such as T cell activation and regulation of lymphocyte activation (Additional file 1: Fig. S2). As a result, 17 drugs were predicted to reserve these trajectories and recover immune status by targeting their DEGs. After obtaining the various drug responses of the trajectories, a bipartite graph model was built and solved. For patient su008, entinostat, a benzamide histone deacetylase inhibitor targeting HDAC1 and HDAC3 treated as monotherapy [79] or combinatorial drug together with immunotherapy to various cancers [80, 81], was ranked 4th in comboSC prediction results as a drug that can be paired with immunotherapy. Also, a recent study reported the role of entinostat in inducing changes in multiple myeloid cell types, reducing immunosuppression, increasing antitumor immune responses, and improving sensitivity to immunotherapy for HER2^+^ breast cancer [82], which indicated the combinatorial effects of entinostat with immunotherapy for middle immune score samples.

Next, we applied comboSC to 44 tumor samples with a middle immune score, and treatment regimens that could be combined with immunotherapy were predicted by comboSC. Interestingly, almost all the small molecules we screened from CMap [10] by comboSC were associated with immunotherapy, and most of them had a function of improving the immune microenvironment. For example, comboSC found the trajectory of active T cell exhaustion and memory T cell exhaustion in the sample Endo2 from GSE139555. Imatinib mesylate (STI-571), which enhances the activation of naive antigen-specific T cells and restores the responsiveness of tolerant T cells from tumor-bearing hosts [83], was one of the top predictions (ranked 2nd) by comboSC. For sample P1 in GSE117570, comboSC found the immune trajectory of TAM differentiation, the gene signature of which is highly similar to the estrone pathway. Antiestrogens combined with immunotherapy that have been found to have the function of the M1 phenotype of TAM polarization and increase immunotherapy response can be used for the treatment of this patient [84]. Furthermore, for sample T15 in CRA001160, comboSC identified a T cell exhaustion trajectory as most T cells are exhausted (Fig. 4g), and ivermectin was ranked 1st in comboSC predictions. We further analyzed the personalized mechanism of the sample that can be intervened by ivermectin in this case. Firstly, comboSC identified the differential expressed genes of the T cell exhaustion trajectory in this patient (Fig. 4h). Secondly, referring to the LINCS database, we found that the top 1st prediction ivermectin showed a suppressive function to downregulated genes and an activation function to upregulated genes of the T cell exhaustion trajectory (Fig. 4i), indicating the mechanism of ivermectin synergized with immunotherapy by repression of the T cell exhaustion trajectory. Interestingly, two top differential genes, STNM1 and RSG1 (Fig. 4h), are all reported to be related to immunotherapy. STNM1 has been reported as a poor prognostic marker in various cancers and can predict poor clinical outcomes of immunotherapy [85], and RSG1 has been reported as an oncogenic marker for poor immune microenvironment [86]. In addition, as an FDA-approved anti-parasitic drug, a recent study reported that ivermectin can induce immunogenic cancer cell death (ICD) and robust T cell infiltration, convert cold tumors into hot, and synergize with immune checkpoint blockade for the treatment of breast cancer [87]. For sample P5 in GSE150660, comboSC identified two immune trajectories as TAM differentiation and active T cell exhaustion, and predicted fluvastatin as one of the top drug predictions. We further analyzed the personalized mechanism of the sample that can be intervened by fluvastatin in this case. Among differential genes of TAM differentiation, hypoxia-inducible factor 1 subunit alpha (HIF1α), a key gene of the hypoxia signaling pathway, was reported as direct target of fluvastatin [88] and regulated the TAM differentiation by cell hypoxia [89]. Another two upregulated genes of the active T cell exhaustion trajectory, UBB and PSM1, were also functional in the pathway of cell response of hypoxia. The top drug prediction fluvastatin identified by comboSC, reported to augment immunotherapy by suppressing the hypoxic tumor environment [90], indicates that the mechanism of synergized drug effect with immunotherapy lies in reducing cell hypoxia response as well as TAM differentiation and activating T cell exhaustion. Collectively, these mechanism analyses provide further support for our predictions.

Furthermore, we also validated comboSC drug predictions for middle immune score patients with the ClinicalTrials.gov database, as in the aforementioned study, and found significant enrichment of the top drugs predicted by comboSC registered in the ClinicalTrials.gov database (Fig. 4i). These recommended perturbations with a function of improving immunity demonstrated the effectiveness of comboSC for middle immune level patients. A detailed summary of all these samples can be accessed in Additional file 2: Table S2.

Finally, we evaluated the predictions of comboSC drug ranking in vivo using pretreatment samples which have been treated with ICB combinatory therapy. We first applied comboSC to a single-cell RNA-seq dataset (SCP1288) with three RCC patients treated with combinatory therapy of ICB and tyrosine kinase inhibitors (TKI) imatinib [91], a drug that inhibits Treg cells and augments immunity in tumor [92], while only P55 showed response to the combinatory therapy. ComboSC correctly classified P55 as a middle immune score, and successfully identified imatinib in comboSC top predictions (ranked 13th in 1288 candidates), which was consistent with the origin study. We next used comboSC for another single-cell RNA-seq dataset (GSE169246) with 6 TNBC patients treated with combinatory therapy of ICB and paclitaxel [65]. We also found higher (*p* < 0.05) comboSC scores of paclitaxel in responders for combinatory therapy than those non-responders (Additional file 1: Fig. S3), indicating the potential of comboSC for predicting optimal personalized drugs for combinatory ICB therapy.

### ComboSC web server

**Fig. 5:**
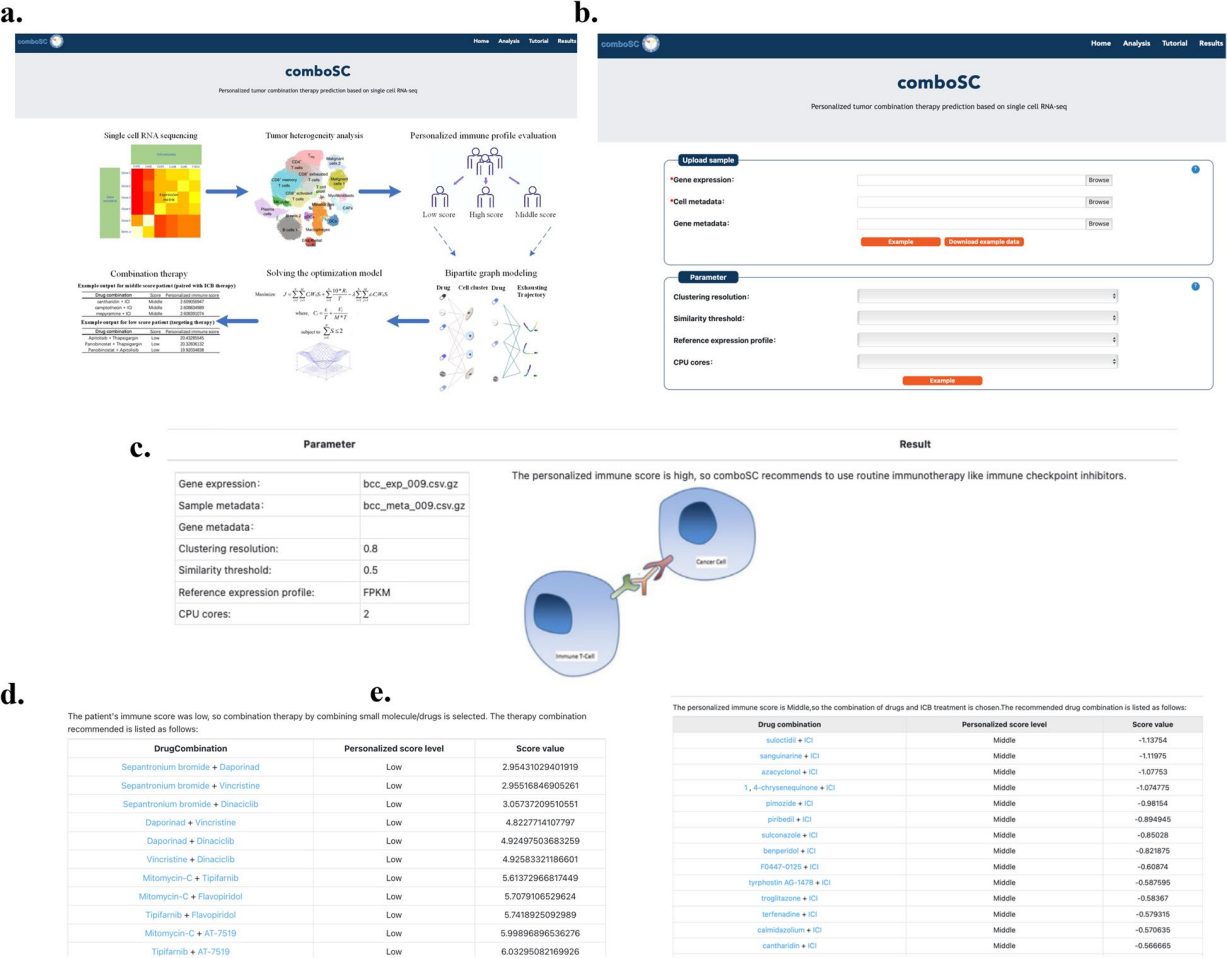
Demo of ComboSC web server. **a** The homepage of the comboSC web server. **b** The data analysis page for the user, with the input of expression matrix, cell metadata, gene metadata, and parameters. **c** The demo output of high immune score samples. **d** The demo output for low immune score samples. Drug combinations in the first column are ranked by the score in the third column. **e** The demo output for middle immune score samples. The numbers in the first column are PubChem CIDs and are ranked by the score in the third column

To facilitate the broad utility of comboSC for clinical and research users, we developed an interactive web server for the quick application of comboSC with tumor scRNA-seq data as input (Fig. 5a). All users need to do is upload the gene expression matrix along with the cell metadata matrix and the gene metadata matrix obtained by single-cell RNA-seq of the tumor sample (Fig. 5b). Then, comboSC will run and complete all the tasks in the backend and finally provide the predicted combination therapy results to the users (Fig. 5c–e). Such a one-stop platform is expected to greatly facilitate personalized tumor treatment at single-cell granularity, serving as a complementary drug screening technique to those of in vivo and ex vivo experimental screenings like PDXs, organoids, etc.

## Discussion

scRNA-seq enables the identification of tumor heterogeneity from different patients with the same disease and different cells in the same patient. We introduced a computational prototype, comboSC, that performs tumor combinatorial therapy optimization based on personalized scRNA-seq data. Taking advantage of the single-cell technique, comboSC first evaluates the immune microenvironment of the tumor sample by the immune score of the Tres model and then matches the immunotherapy or target therapy to the individual immune score of the tumor sample. Next, for tumor samples that are not suitable for direct immunotherapy, comboSC uses a bipartite graph model to optimize the combinatorial therapy response. Specifically, for tumor samples with low immune scores (no immunotherapy response), comboSC performs drug combinatorial optimization for the response to complex cell groups in vivo. For tumor samples with medium immune scores (partial immunotherapy response), comboSC uses an immune recovery strategy and performs bipartite graph modeling of the four selected immune-related trajectories, considering the current status with RNA expression and future trends with RNA velocity, to select small molecules that can be paired with ICB to boost immunotherapy. Finally, comboSC provides therapy combinations ranked by response score. The whole pipeline of comboSC could be easily applied to customized data with comboSC online web server.

Although we applied comboSC to various tumor samples and tumor types, there are still limitations in the current version of comboSC. First, due to the limited dataset sizes for rare cancer types used in the building of comboSC, the application of comboSC for rare cancer types should be carefully investigated. Second, comboSC only analyzes four immune exhaustion trajectories, which is likely an oversimplification of complex immune exhaustion mechanisms. More immune exhaustion pathways should be considered with future discoveries from the tumor immune microenvironment. Thirdly, comboSC optimized the drug response to different cell groups, however neglected the simultaneous effect of targeted drugs on both tumor and immune cells. Several future updates of comboSC are expected, including expansion for broader application with emerging tumor single-cell datasets and extension for new tumor immune-related mechanisms.

It should be noted that comboSC can be used as an efficient screening framework to predict potential drug combination therapies for personalized tumor treatment; however, further large-scale experimental validation using various model systems, including PDXs or organoids, is needed. Nevertheless, comboSC will greatly accelerate the whole personalized treatment procedure, reducing screening time from a large drug combination space and saving the valuable treatment time of patients, with the increasing cost reduction of single-cell sequencing. In summary, comboSC is a novel and inspired prototype for personalized microenvironment evaluation and tumor combinatorial therapy optimization at single-cell granularity. The rationale of comboSC as well as the developed web server of comboSC is expected to greatly facilitate personalized clinical tumor therapy and precision oncology with the integration of the underlying information from the personalized cell landscape.

## Conclusions

In summary, comboSC offers a practical and comprehensive computational prototype, along with a proof-of-concept study, designed to predict potential drug combinations. These predictions can then be further validated through experiments and applied in clinical settings using single-cell transcriptome data. This approach aims to streamline and expedite personalized tumor treatment by minimizing screening time within the vast drug combination space. As a result, valuable treatment time for individual patients can be saved.

### Supplementary Information

**Additional file 1: Fig. S1.** The strategy of the comboSC drug-cell cluster response prediction. **Fig. S2.** GO enrichment bubble plot of differential genes in immune exhaustion trajectory. **Fig. S3.** ComboSC score of paclitaxel in responders and non-responders to combinatory therapy in GSE169246 dataset.

**Additional file 2: Table S1.** Summary of tumor single cell RNA-seq datasets. **Table S2.** Details of tumor single cell RNA-seq datasets and comboSC results. **Table S3.** Details of drugs from GDSC database used in comboSC. **Table S4.** Details of drugs from CMAP database used in comboSC. **Table S5.** Tres score predictions and immunotherapy responses of all datasets in Fig. 2e-i. **Table S6.** Top 50 comboSC predictions for low and middle immune score samples. **Table S7.** ICB combinatory treatment sample information.

## Data Availability

The accession number and sample information of all datasets analyzed in this study are listed in Additional file 2: Table S2. The included samples are as follows: the human AML dataset was obtained from Gene Expression Omnibus (GEO), with accession numbers GSE116256 (https://www.ncbi.nlm.nih.gov/geo/query/acc.cgi?acc=GSE116256) [93] and GSE154109 (https://www.ncbi.nlm.nih.gov/geo/query/acc.cgi?acc=GSE116256) [94]. Data concerned with BCC was available at the GEO repository: GSE123813 (https://www.ncbi.nlm.nih.gov/geo/query/acc.cgi?acc=GSE123813) [72]. The BRCA datasets were obtained from GEO repository: GSE143423 (https://www.ncbi.nlm.nih.gov/geo/query/acc.cgi?acc=GSE143423) [69] and GSE176078 (https://www.ncbi.nlm.nih.gov/geo/query/acc.cgi?acc=GSE176078) [95]. The dataset of CHOL was downloaded from GEO under the accession code GSE125449 (https://www.ncbi.nlm.nih.gov/geo/query/acc.cgi?acc=GSE125449) [96] and GSE138709 (https://www.ncbi.nlm.nih.gov/geo/query/acc.cgi?acc=GSE138709) [97]. The human CRC datasets were downloaded from the GEO repository: GSE139555 (https://www.ncbi.nlm.nih.gov/geo/query/acc.cgi?acc=GSE139555) [98] and GSE146771 (https://www.ncbi.nlm.nih.gov/geo/query/acc.cgi?acc=GSE146771) [99]. The human HNSC dataset was obtained from GEO, with accession number GSE103322 (https://www.ncbi.nlm.nih.gov/geo/query/acc.cgi?acc=GSE103322) [100]. The human KIRC datasets were downloaded from the GEO repository: GSE121636 (https://www.ncbi.nlm.nih.gov/geo/query/acc.cgi?acc=GSE121636) [101]. Data concerned with LIHC was available at the GEO repository: GSE139555 (https://www.ncbi.nlm.nih.gov/geo/query/acc.cgi?acc=GSE139555) [98] and GSE140228 (https://www.ncbi.nlm.nih.gov/geo/query/acc.cgi?acc=GSE140228) [23]. The NSCLC datasets were obtained from the GEO repository: GSE127465 (https://www.ncbi.nlm.nih.gov/geo/query/acc.cgi?acc=GSE127465) [102], GSE139555 (https://www.ncbi.nlm.nih.gov/geo/query/acc.cgi?acc=GSE139555) [98], GSE143423 (https://www.ncbi.nlm.nih.gov/geo/query/acc.cgi?acc=GSE143423) [69], and GSE150660 (https://www.ncbi.nlm.nih.gov/geo/query/acc.cgi?acc=GSE150660) [103]. The human OS datasets were downloaded from the GEO repository: GSE162454 (https://www.ncbi.nlm.nih.gov/geo/query/acc.cgi?acc=GSE162454) [104]. The human PAAD datasets were downloaded from the Genome Sequence Archive: CRA001160 (https://ngdc.cncb.ac.cn/gsa/browse/CRA001160) [105]. Data concerned with SKCM was available at the GEO repository: GSE115978 (https://www.ncbi.nlm.nih.gov/geo/query/acc.cgi?acc=GSE115978) [100]. The SKCM datasets were obtained from the GEO repository: GSE115978 (https://www.ncbi.nlm.nih.gov/geo/query/acc.cgi?acc=GSE115978) [100], GSE118056 (https://www.ncbi.nlm.nih.gov/geo/query/acc.cgi?acc=GSE118056) [106], and GSE72056 (https://www.ncbi.nlm.nih.gov/geo/query/acc.cgi?acc=GSE72056) [107]. Data concerned with SS was available at the GEO repository: GSE131309 (https://www.ncbi.nlm.nih.gov/geo/query/acc.cgi?acc=GSE131309) [108]. Data concerned with THCA was available at the GEO repository: GSE148673 (https://www.ncbi.nlm.nih.gov/geo/query/acc.cgi?acc=GSE148673) [52]. The human UCEC dataset was obtained from GEO, with accession number GSE139555 (https://www.ncbi.nlm.nih.gov/geo/query/acc.cgi?acc=GSE139555) [98]. The human UVM dataset was obtained from GEO, with accession number GSE139829 (https://www.ncbi.nlm.nih.gov/geo/query/acc.cgi?acc=GSE139829) [28]. The comboSC top50 drug predictions for all analyzed samples are listed in Additional file 2: Table S6. The details of samples treated with ICB combinatory therapy are listed in Additional file 2: Table S7. Specifically, the human RCC and TNBC datasets were acquired from the Single Cell Portal under accession number SCP1288 (https://singlecell.broadinstitute.org/single_cell/study/SCP128) and from the Gene Expression Omnibus under accession number GSE169246 (https://www.ncbi.nlm.nih.gov/geo/query/acc.cgi?acc=GSE169246) [65]. The comboSC tools can be accessed from the web server (www.comboSC.top), and the source code is available at GitHub (https://github.com/bm2-lab/comboSC).

## Abbreviations

AML: Adult acute myeloid leukemia
AUROC: Area under the receiver operating characteristic
BCC: Basal cell carcinoma
BRCA: Breast invasive carcinoma
CAF: Cancer-associated fibroblast
CCLE: Cancer Cell Line Encyclopedia
CMap: Connectivity Map
CR: Complete response
CRC: Colorectal cancer
DEG: Differentially expressed gene
GDSC: Genomics of Drug Sensitivity in Cancer
HNSC: Head and neck cancer
ICB: Immune checkpoint blockade
ICD: Immunogenic cancer cell death
KIRC: Kidney renal clear cell carcinoma
LIHC: Liver hepatocellular carcinoma
NR: No response
NSCLC: Non-small cell lung cancer
OS: Osteosarcoma
PAAD: Pancreatic adenocarcinoma
PD-1: Programmed cell death-1
PDCs: Patient-derived cell lines
PD-L1: Programmed death-ligand 1
PDX: Patient-derived tumor xenograft
PR: Partial response
scRNA-seq: Single-cell RNA sequencing
SKCM: Skin cutaneous melanoma
SS: Synovial sarcoma
TAM: Tumor-associated macrophage
THCA: Thyroid carcinoma
TKI: Tyrosine kinase inhibitors
TME: Tumor microenvironment
Tres: T cell resilience
UCEC: Uterine corpus endometrioid carcinoma
UVM: Uveal melanoma
