# Supplementary material for: Personalized tumor combination therapy optimization using the single-cell transcriptome
Source: Genome Med. 2023 Dec 1;15:105. doi: 10.1186/s13073-023-01256-6 (PMC10691165; doi:10.1186/s13073-023-01256-6)
Supplement: Supplementary file 1 — Additional file 1: Fig. S1. The strategy of the comboSC drug-cell cluster response prediction. Fig. S2. GO enrichment bubble plot of differential genes in immune exhaustion trajectory. Fig. S3. ComboSC score of paclitaxel in responders and non-responders to combinatory therapy in GSE169246 dataset. [file 13073_2023_1256_MOESM1_ESM.pdf]

Personalized tumor combination therapy optimization using single cell transcriptome

Additional file 1: Supplementary Figures

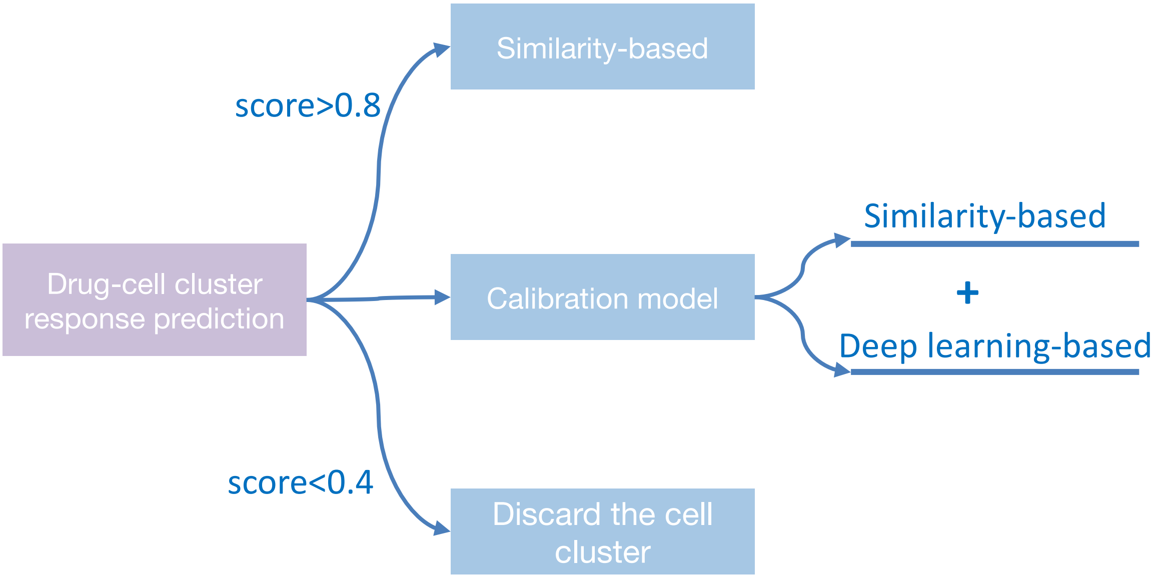

Fig. S1 The strategy of the comboSC drug-cell cluster response prediction. ComboSC takes different methods based on the similarity score. If the similarity score is over 0.8, comboSC will use the similarity model. If the similarity score is lower than 0.4, comboSC will neglect the cell. If the similarity score is higher than 0.4 but lower than 0.8, comboSC will take a calibration model incorporating both similarity and deep learning model.

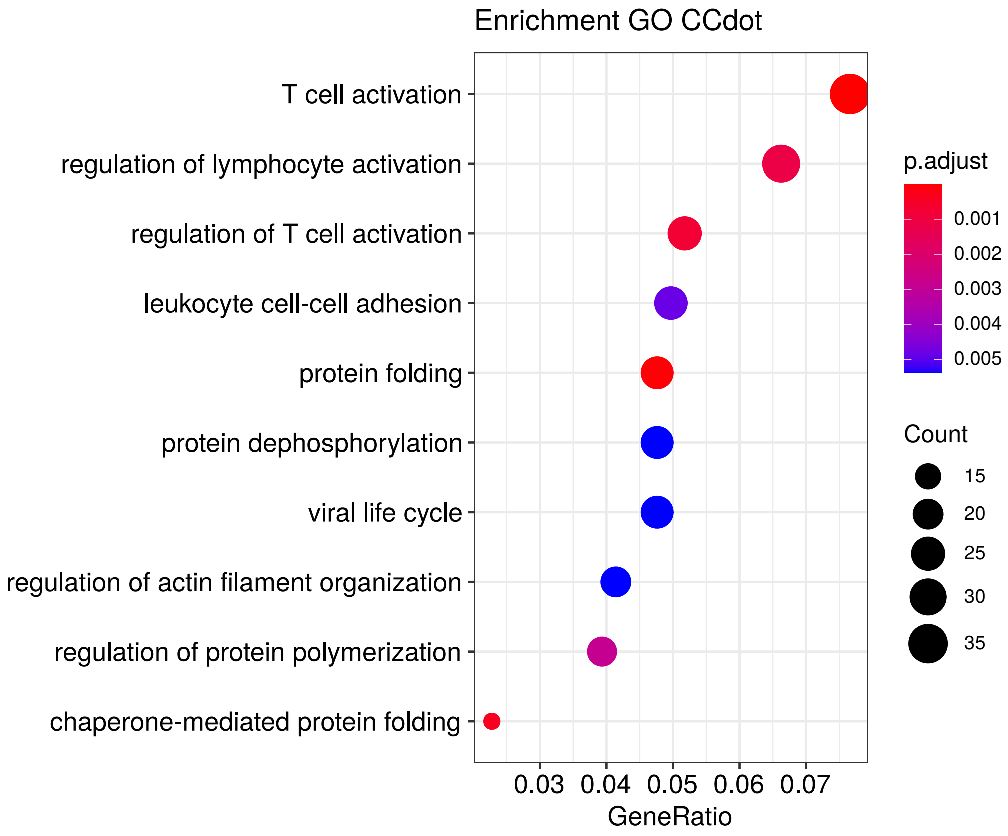

Fig. S2 GO enrichment bubble plot of differential genes in immune exhaustion trajectory.

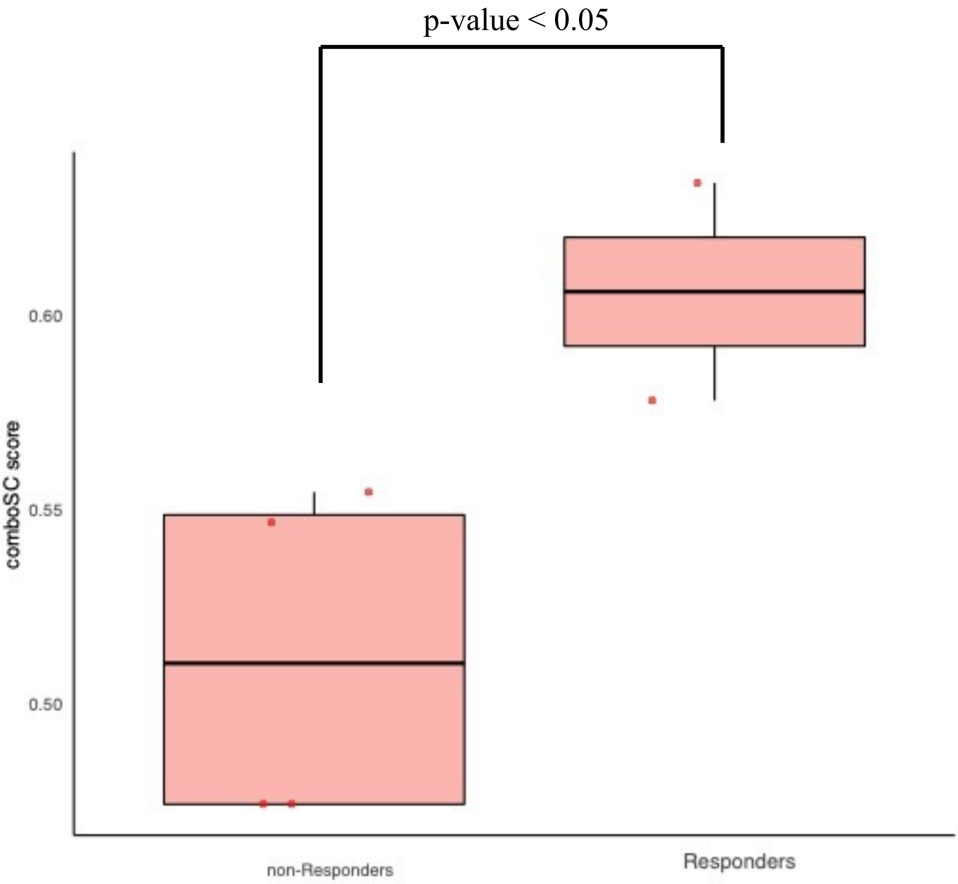

Fig. S3 ComboSC score of paclitaxel in responders and non-responders to combinatory therapy in GSE169246 dataset.
